# Delay Discounting in Gambling Disorder: Implications in Treatment Outcome

**DOI:** 10.3390/jcm11061611

**Published:** 2022-03-14

**Authors:** Teresa Mena-Moreno, Giulia Testa, Gemma Mestre-Bach, Romina Miranda-Olivos, Rosario Granero, Fernando Fernández-Aranda, José M. Menchón, Susana Jiménez-Murcia

**Affiliations:** 1 Department of Psychiatry, University Hospital of Bellvitge, L’Hospitalet de Llobregat, 08907 Barcelona, Spain; tmena@bellvitgehospital.cat (T.M.-M.); gtesta@idibell.cat (G.T.); rmiranda@idibell.cat (R.M.-O.); ffernadez@bellvitgehospital.cat (F.F.-A.); jmenchon@bellvitgehospital.cat (J.M.M.); 2 Ciber Fisiopatología Obesidad y Nutrición (CIBERObn), Instituto de Salud Carlos III, 28029 Madrid, Spain; roser.granero@uab.cat; 3 Facultad de Ciencias de la Salud, Universidad Internacional de La Rioja, La Rioja, 26006 Logroño, Spain; gemma.mestre@unir.net; 4 Department of Psychobiology and Methodology, Autonomous University of Barcelona, 08193 Barcelona, Spain; 5 Psychoneurobiology of Eating and Addictive Behaviors Group, Neurosciences Programme, Bellvitge Biomedical Research Institute (IDIBELL), 08908 Barcelona, Spain; 6 Psychiatry and Mental Health Group, Neuroscience Program, Institut d’Investigació Biomèdica de Bellvitge-IDIBELL, L’Hospitalet de Llobregat, 08907 Barcelona, Spain; 7 Ciber Salut Mental (CIBERSAM), Instituto de Salud Carlos III, 28029 Madrid, Spain

**Keywords:** impulsivity, delay discounting, gambling disorder, treatment outcomes, gambling preferences

## Abstract

Impulsive choice, measured by delay discounting (DD) tasks, has been shown in patients with gambling disorders (GD). However, the impact of DD and treatment outcome has been scarcely explored in GD patients. The aims of this study were: (1) to examine the baseline association between DD and clinical variables in GD patients depending on their age and gambling preferences (strategic vs. non-strategic); and (2) to estimate the predictive role of DD on poorer outcomes of cognitive-behavioral therapy (CBT) when considering also the effect of other clinical variables. 133 treatment-seeking male GD patients were evaluated at baseline with a DD task and measures of GD severity, personality traits and psychopathology. Treatment outcome was measured in terms of dropout from CBT and relapses. Results showed baseline associations between DD and GD severity (correlation coefficient R = 0.408 among strategic gamblers and R = 0.279 among mixed gamblers) and between DD and positive/negative urgency (R = 0.330 for the youngest patients, R = 0.244 for middle age, and around R = 0.35 for gamblers who reported preferences for strategic games). Other personality traits such as high harm avoidance and low cooperativeness were also related to DD at baseline (R = 0.606 among strategic gamblers). Regarding treatment outcome, a steeper discount rate predicted a higher risk of relapses in strategic gamblers (odds ratio OR = 3.01) and middle-age ones (OR = 1.59), and a higher risk of dropout in younger gamblers (OR = 1.89), non-strategic gamblers (OR = 1.70) and mixed gamblers (R = 4.74). GD severity mediated the associations between age, DD, personality traits and poor CBT outcome. In conclusion, impulsive choice affects treatment response in individuals with GD and may interfere with it to a significant extent. Considering DD in GD, patients seeking treatment could help control its impact on treatment adherence and relapses.

## 1. Introduction

Gambling Disorder (GD) is characterized by a persistent and recurrent urge to gamble that causes clinical distress or impairment in family, professional or personal areas. It is considered a multicausal behavioral addiction in which different psychological, biological and environmental factors intervene and interact [1]. GD usually begins in adolescence or young adulthood, but in some individuals it manifests in middle adulthood or at a more advanced age [2]. Gender differences are also present, with higher vulnerability of male adolescents to gambling [3], and a higher prevalence of GD in male adults [4,5].

High impulsivity is a core characteristic of GD [6,7,8,9,10] and it is expected to impact treatment outcomes. Impulsivity is increasingly understood as a multidimensional construct that includes multiple dimensions [11,12,13]. In terms of personality traits, the UPPS [14] is a widely adopted model which divides impulsivity into four dimensions: (i) lack of premeditation, involving acting without thinking; (ii) lack of perseverance, representing the tendency to not finish tasks; (iii) sensation seeking, encompassing behavior tendencies to try new and exciting activities or sensations; (iv) as well as positive and negative urgency and a tendency to act rashly in response to extreme negative or positive emotions. This is a validated model across different age samples and from which the UPPS-P Scale was developed [14].

Another dimension of impulsivity is impulsive choices measured with a delay discounting (DD) task [15]. This refers to the extent to which the subjective value of a reward decreases as the delay to receipt increases. It is commonly assessed through DD tasks where individuals have to choose between immediate and delayed rewards (e.g., money), scored by calculating the respondent’s discount rate (k) or other quantitative indices (e.g, area under the curve, impulsive choice ratio). A steeper discount rate and, subsequently, a smaller area under the discounting curve is frequently interpreted as an impulsive preference for immediate over delayed rewards [16,17,18].

Several studies have found higher discount rates in patients with GD than healthy controls [15,19,20,21,22,23]. Moreover, there is evidence of an association between the severity of GD symptoms and high discount rates [22,24]. Steeper discounts of delayed rewards in patients with GD is associated with greater risk-taking, poorer decision-making, higher levels of bet chasing [25], and impulsivity traits [26,27,28]. Similarly, other personality traits frequently linked to GD severity that represent impulsive attitudes (e.g., high novelty seeking, low self-directedness) [29,30,31] might present associations with discount rates, although their relationship with DD has not been explored yet.

Therapies based on cognitive-behavioral approaches are considered as an effective treatment for GD, although there are other therapies successfully adopted, alone or in combination with CBT, including motivational interventions [32,33,34]. It has been shown that high baseline impulsivity is correlated with lower adherence to treatment and worse outcomes in patients with GD [35,36,37,38,39]. As for personality traits, lack of perseverance and positive urgency were related to dropout from treatment, whereas negative urgency was related to a higher number of relapses [35,40]. Moreover, impulsive choice (measured through the DD task) predicted therapy dropout and relapses in other disorders such as substance abuse [41,42,43,44,45]. Still, the relation between impulsive choice and treatment outcome has not been robustly explored in GD. To date, there is only a study that showed no direct effect between DD and the quantity of money wagered over time nor the abstinence from gambling [46]. Nevertheless, no studies have investigated whether DD could be related to treatment adherence and short-term relapse following cognitive-behavioral therapy (CBT).

Furthermore, DD may impact treatment response, especially in those patients presenting specific personality traits or with more severe symptomatology. Previous studies suggested that impulsivity-related personality traits seem to characterize certain profiles of gamblers, especially those with strategic preferences (e.g., dice, poker, and other cards, betting on sports events or races, or the stock market) [47]. In non-clinical populations, age is another factor affecting discount rates, where more impulsive choices are observed in younger individuals [48,49]. Overall, younger gamblers have been described as more impulsive than the oldest ones [26,50]. Thus, discount rates could affect responses to treatment differently in younger than older patients.

This research aimed to: (1) examine the association between individual discount rates and the clinical profile at baseline (e.g., GD severity, general psychopathology and personality traits) in different age groups and gambling preferences (strategic vs non-strategic); (2) examine whether DD predicts relapse and dropout risk; and (3) to estimate direct and indirect associations between discount rates, GD severity, and personality traits on poor treatment outcome. We hypothesize that higher discount rates would be associated with worse treatment outcomes (relapses, dropouts), especially in younger patients (vs. older patients) and strategic gamblers (vs. non-strategic patients). These groups would show higher risks of dropout and relapses as a function of discount rates. Finally, in patients with higher discount rates, more severe GD symptoms and more impulsive personality traits would have poorer treatment outcomes.

## 2. Materials and Methods

### 2.1. Participants

The sample consisted of 133 consecutive patients voluntarily seeking treatment for GD, recruited between December 2018 and November 2020 at the Behavioral Addictions Unit within the Department of Psychiatry at Bellvitge University Hospital (Barcelona, Spain). Inclusion criteria were being diagnosed with GD, previously screening via methods (SOGS, DSM-5 criteria and other relevant measures to identify psychopathological symptoms) and confirmed by individual structured interviews by experienced clinical psychologists and psychiatrists with more than 20 years of experience in the field. Exclusion criteria included: (a) the presence of a psychiatric or neurological disorder such as schizophrenia or other psychotic disorders that might impact game performance, (b) an intellectual disability, and (c) any active pharmacological therapy that might interfere.

All participants received information regarding the aims of the research, and they provided signed informed consent for participating. There was no financial or other compensation for being part of the study. Participants who agreed to take part in the research were briefed on the purpose of the study and were reassured of the voluntary nature of their participation and their rights to stop at any time. The study was approved by the Ethics Committee of the first author’s hospital (ref. number PR286/14), adhering to the principles outlined in the latest version of the Declaration of Helsinki.

### 2.2. Procedure

All participants underwent the same baseline assessment of impulsive choices, GD severity, general psychopathological symptoms and personality traits. After baseline evaluation, all patients received treatment with a standardized 16-week cognitive-behavioral therapy (CBT) intervention described in previous works [51]. The goal of the treatment was to train patients to implement CBT strategies in order to minimize gambling maladaptive behaviors. The outcome of the treatment was measured by treatment dropout and relapses. A relapse indicates that the patients present a full gambling episode. Failure to attend three consecutive CBT sessions was considered a criterion for dropout.

### 2.3. Baseline Assessment

#### 2.3.1. Delay Discounting Task

DD was assessed using a validated paper-and-pencil task [16] consisting of 27 items that provide a set of choices between a smaller immediate monetary reward and a larger delayed monetary reward. Each item corresponds to a different k value that represents the amount of discounting of the later reward that renders it equal to the smaller reward. K-values can range from 0 (selection of the delayed reward option for all items, or no discounting) to 0.25 (selection of the immediate reward option for all items, or always discounting). The respondent’s answers permit the calculation of their discounting curve, with steeper curves indicating higher levels of impulsivity. Given the hyperbolic distribution of discount rate values [52], an individual’s discount rates were normalized using natural logarithmic (ln) transformation to calculate the natural log transformation (nlog k-values) method used in previous studies [26,52,53,54].

#### 2.3.2. Gambling Disorder Diagnosis and Severity

The Diagnostic Questionnaire for Pathological Gambling [55] is a 19-item diagnostic questionnaire based on DSM-5 criteria [56] designed to measure the presence of the GD diagnosis (present-absent) and the level of severity (zero criteria: non-problem gambling; 1–3 criteria, problem gambling; 4–5 criteria, moderate-GD; 6–7 criteria, mild-GD; 8–9 criteria, severe-GD). The sum of the DSM-5 criteria/symptoms was adopted as a measure of the GD severity within a continuum ranging from 4 to 9, as in previous studies [57]. The Spanish adaptation of the questionnaire was used in this study (Cronbach’s alpha α = 0.81 for the general population and α = 0.77 for clinical samples; [58]. The internal consistency for this scale in the study sample was good (α 0.75).

The South Oaks Gambling Screen (SOGS) [59]: This is a self-report 20-item questionnaire to ascertain gambling disorder severity. This screening questionnaire discriminates between probable pathological, problem, and non-problem gamblers. The Spanish validation used in this work [60] showed excellent internal consistency (α = 0.94), convergent validity (R = 0.92), and test-retest reliability (r = 0.98) [60]. The internal consistency in the study sample was in the good range (α = 0.843).

#### 2.3.3. Impulsive Behavior Scale (UPPS-P)

The UPPS-P questionnaire [9] is composed of 59 items, measuring five dimensions of impulsive personality traits through self-report on 59 items: negative urgency; positive urgency; lack of premeditation; lack of perseverance; and sensation seeking. The Spanish-language adaptation shows good reliability (Cronbach’s α between 0.79 and 0.93) and external validity [61]. Consistency in the study sample was between good (α = 0.75 for lack of perseverance scale) to excellent (α = 0.92 for positive urgency).

#### 2.3.4. Temperament and Character Inventory-Revised (TCI-R)

The TCI-R questionnaire [54] contains 240 items for measuring personality traits structured in seven personality dimensions: four of them are related to temperament (novelty seeking, harm avoidance, reward dependence, and persistence). Consistency for each dimension in our sample was α = 0.750, α = 0.841, α = 0.795, α = 0.900, respectively, and three were character dimensions (self-directedness, cooperativeness and self-transcendence). Consistency for each dimension in our sample was α = 0.899, α = 0.863, α = 0.859, respectively). For the current study, the Spanish version of TCI-R was used [62].

#### 2.3.5. Symptom Checklist-Revised (SCL-90-R)

The SCL-90 [63] self-report tool measures the global psychological state through 90 items structured in nine primary dimensions: There are three global indices that were adopted in the study: GSI (global severity index), PST (positive symptoms total), and PSDI (positive symptoms discomfort index). The Spanish version of this questionnaire has obtained good to adequate indices (mean α = 0.75) [64]. Internal consistency for all of the global indices in this study sample was 0.978.

#### 2.3.6. Other Sociodemographic and Clinical Variables

Additional demographic, clinical, and social/family variables related to gambling were measured using a semi-structured face-to-face clinical interview described elsewhere [51]. This interview explores several variables related to gambling behavior, such as the maximum bet in a single gambling episode, the average amount of money spent in each episode, the motives for gambling (emotion regulation, search for prizes, etc.), debts, illegal acts, attitudes of the family in front of the problem, etc.

### 2.4. Statistical Analysis

A statistical analysis was carried out with Stata17 for Windows [65]. Correlation models were used to assess the association between the delay discounting score and the clinical state at baseline. Estimates were obtained within the total sample and also stratified by the groups defined by the participants’ age. Three sub-groups based on other studies within the GD area were considered [50,57,66,67]: younger age (18 to 34 years old), middle-age (35 to 50 years old), and older age (51 to 80 years)], in addition to the gambling preference (non-strategic, strategic and mixed). Partial correlations adjusted by age were obtained within the groups of patients with different gambling preferences. In this work, due to the strong association between the null-hypothesis test for the correlation models with the sample size, only R-coefficients within the range of mild-medium (|R| > 0.24) to high-large (|R| > 0.37) were considered as relevant [68].

## 3. Results

### 3.1. Characteristics of the Sample

The sample consisted of *N* = 133 male patients with a mean age of *M* = 44.6 years old (*SD* = 12.7). Most participants in the study achieved primary (*n* = 70, 52.6%) or secondary (*n* = 44, 33.1%) education levels (the remaining *n* = 19 participants reported tertiary level). Most participants were also married or single (married: *n* = 76, 57.1%; single: *n* = 42, 31.6%; divorced or separated: *n* = 15, 11.3%), pertained to mean low or low social position indexes (*n* = 107, 80.5%), and were employed (*n* = 72, 54.1%).

Regarding the GD-related measures, the mean age of onset of the gambling problems was 30.0 years old (*SD* = 10.5), and the mean duration of the problematic gambling activities was 6.1 years (*SD* = 6.0). The most prevalent gambling preference was non-strategic games (*n* = 96, 72.2%), followed by strategic games (*n* = 18, 13.5%) and mixed games (*n* = 19, 14.3%). The number of participants who reported debts due to gambling activity was *n* = 54 (40.6%).

Considering the results of the global indexes of the SCL-90 R at baseline, the number of participants within the clinical range for the GSI was *n* = 80 (60.2%), for the PST *n* = 84 (63.2%) and for the PSDI *n* = 22 (16.5%). For the impulsive dimensions measured with the UPPS-P, the prevalence of participants within the clinical range was between 6% (for the sensation seeking scale) and 29.3% (for the positive urgency scale).

### 3.2. Association between Delay Discounting and Clinical Profile at Baseline

Table 1 displays the correlation matrix between the delay discount rates and the clinical profile at baseline (partial correlation is adjusted by the participants’ age). Bold values indicate effect sizes within the mild-moderate to high-large ranges. While no relevant association was found within the total sample, results stratified by the groups of age and the gambling preference evidenced that: (a) among the younger age individuals, delay discount rates positively correlated with the chronological age, the duration of the GD, the SOGS-total, and the positive and negative urgency scores, while negative correlations were found with the age of onset, the hostility and the phobic anxiety levels; (b) among the middle age group, delay discounting positively correlated with the GD severity (number of DSM-5 criteria and SOGS-total), the positive urgency and harm avoidance, and negatively with the cooperativeness level; (c) within the non-strategic gamblers, delay discount rates were negatively associated with the anxiety levels; (d) within the strategic gamblers, positive associations were found between delay discount rates and age, GD severity levels, psychopathology levels (SCL-90R scales, except for somatization) impulsivity (UPPS-P scales, except for sensation seeking), and harm avoidance, while negative correlations with persistence, self-directedness, cooperativeness and self-transcendence were observed; and (e) within the individuals who reported mixed gambling preference, delay discount rates positively correlated with age, GD severity and impulsivity (except with lack of perseverance and sensation seeking), and negatively correlated with the SCL-90R PSDI and self-directedness.

### 3.3. Association between Delay Discounting and CBT Outcomes at Baseline

The number of patients who abandoned the CBT was n = 25 (risk of dropout equal to 18.8%), and the number of patients who reported the presence of gambling episodes was n = 39 (risk of relapses equal to 29.3%).

Table 2 shows the coefficients that measure the association of the delay discount rates and the CBT outcomes in the study (dropouts and relapses). The associations between delay discounting with risk of dropout and relapse were estimated with odds ratio (OR) coefficients, while partial correlations estimated the associations between delay discounting and the number of treatment sessions the patients attended, the number of relapses during the CBT and the euros spent during the relapses. All the estimates were adjusted by the patients’ age. Within the total subsample, delay discount rates were positively related to the risk of dropout (OR = 1.43). Considering the groups defined by age and the gambling preference, and for the delay discounting measure, the next associations were found: (a) with the risk of dropout within the younger age patients, the non-strategic and the mixed gamblers; (b) the risk of relapse within the middle age patients and the strategic gamblers; (c) the number of treatment sessions (with negative correlations) within the younger age patients and the mixed gamblers; (d) the number of relapses and the amount of euros spent in the gambling episodes within the strategic gamblers.

Table 3 shows the results of the two logistic models assessing the specific predictive capacity of the independent variables: age, duration of the GD, GD severity, psychopathology state, impulsivity, gambling preference, and delay discount rates. Both criteria measure the main CBT outcomes considered in this work (risk of dropout and relapses during the treatment). The risk of dropout was associated with younger age, lower GD severity, and higher delay discount rates. The risk of relapses was associated with higher delay discounting scores.

### 3.4. Path-Analysis

**Figure 1:**
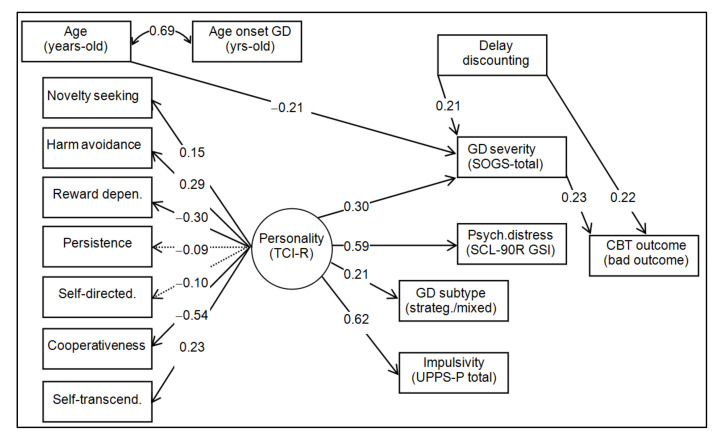
Path-diagram: standardized coefficients. Note. Bad outcome: dropout or relapse. Sample size: n = 133.

Figure 1 shows the path-diagram with the standardized coefficients in the SEM (Table S1, Supplementary Material) and includes the complete results of testing direct, indirect, and total effects. Adequate goodness of fit was obtained: RMSEA = 0.072; CFI = 0.907; TLI = 0.901; SRMR = 0.084.

The latent variable defined by the TCI-R scores achieved significant contributors for all the personality dimensions, except for the persistence and self-directedness. Higher scores in this latent variable were related to higher scores in novelty-seeking, harm avoidance, and self-transcendence, and with lower scores in reward dependence and cooperativeness.

The bad CBT outcome (dropout or relapse) was directly associated with higher levels of delay discounting and GD severity. The GD severity achieved a mediational role in the relationships between delay discounting, personality, and age and CBT outcome: younger age, higher values in delay discount rates, and higher scores in the personality latent variable predicted higher levels in the GD severity, and the next more severe GD behavior increased the likelihood of a bad CBT outcome. Additionally, although not related to the CBT outcome, higher levels in the personality latent variable also increased worse psychopathological distress, the impulsivity levels, and the probability of strategic or mixed gambling preference.

## 4. Discussion

The present study investigated impulsive choices in patients with GD. Specifically, a delay discounting (DD) task was adopted to explore the relation between discount rates and short-term response to cognitive-behavioral therapy (CBT) in a large sample of male GD patients.

Impulsive choice, indexed by higher discount rates, was associated with higher GD severity at baseline. In younger patients, higher discount rates were also associated with an earliest gambling onset, and with a longer duration of the disorder, which may be considered as other indexes of severity and chronicity. These findings corroborate the evidence for the relation between the severity of GD and impulsivity, which has been demonstrated by studies using multiple measures of impulsivity [69]. Regarding choice impulsivity, more severe GD symptoms have been previously associated with DD [24,70,71,72]. Thus, these results suggest that the tendency to prefer economic rewards in the most immediate way possible rather than receiving larger amounts of money in a more distant time is related to a more severe clinical profile of gamblers, which in turn is expected to impact treatment outcomes.

Furthermore, baseline associations between different impulsivity dimensions emerged, especially in younger gamblers. Specifically, both younger and middle age gamblers with more impulsive choices were characterized by higher negative urgency. In the youngest group of patients, the association with DD was also present for positive urgency. Emotion-laden impulsivity, which is represented by urgency, has been linked to affective mechanisms related to problem gambling. Previous evidence that explored a relationship between trait impulsivity and DD in GD patients also showed a link between urgency and impulsive choice [26,27,28,71]. The present findings confirm that the difficulty to postpone immediate gratification is directly related to emotional impulsivity (i.e., a tendency to act rashly when experiencing emotional states) in young patients with GD. In addition, a steeper discount rate showed a relationship with high levels of harm avoidance and low levels of cooperativeness in middle-aged patients. Harm avoidance is characterized by a greater tendency to anxiety, worry and insecurity, isolation, and disconnection from the environment, poor decision-making skills, and planning skills [73]. Studies showed harm avoidance in more severe GD patients who tend to use gambling as a dysfunctional mechanism to avoid problems and regulate emotions [73]. Therefore, gambling could be a dysfunctional habit acquired and maintained over time as a mechanism to avoid problems and difficulties in emotion regulation. Low cooperativeness is typical of low empathy and self-absorbed individuals who primarily look out for themselves [74]. Thus, it could be that these patients are less prone to recognize their gambling as problematic, despite the external pressure (e.g., family, partner) for seeking treatment.

Another finding emerged from the baseline evaluation due to the decision making profile of patients with preferences for strategic gambling. In this group, higher discount rates correlated with higher severity of GD and higher general psychopathology. More severe clinical profiles have been frequently reported in strategic gamblers, often measured in terms of higher bets, higher levels of psychopathology [73,75], cognitive distortions, and more severe GD symptoms [75,76,77]. Moreover, higher discount rates in strategic gamblers were associated with higher levels of negative urgency and harm avoidance, which are personality traits related to emotional dysregulation. Both impulsivity-related traits and impulsive decision-making have been reported in strategic gamblers [35,73,75], and the present findings further suggest a link between these measures of impulsivity in strategic gamblers. In addition, other maladaptive personality traits showed a relation with impulsive choice in this group, including low cooperativeness, self-direction, persistence, and self-transcendence, which could additionally contribute to a more severe clinical profile [75].

Regarding the relation between DD and treatment outcome, more impulsive choice predicted the worst outcomes, coinciding with previous findings in other addictions [44,78,79]. Differences emerged when considering groups of age and strategic preferences. On the one hand, younger gamblers showed an association between discount rates and higher risk of dropout, attending a lower number of CBT sessions before abandoning treatment. By contrast, the middle age group showed an association between DD and higher risk of relapses. On the other hand, DD predicted a higher risk of dropout in patients with preferences for non-strategic or mixed types of gambling and a higher risk of relapse in those with a preference for strategic games.

The association between DD and dropout has been previously described in substance use disorders, such as alcohol dependence [80]. However, this is the first study showing evidence for an association between DD and low treatment adherence in young GD patients. As for relapses, results coincide with previous studies in the field of substance addictions that also observed that high discount rates, together with other factors related to impulsivity, predicted relapses [79]. Therefore, as suggested by other authors, DD may be considered a neurocognitive risk factor that may have a specific impact on the ability of individuals to complete treatment and to remain abstinent [80]. Likewise, this impact of DD on treatment response would be more evident in younger age GD patients. Furthermore, the co-occurrence of impulsive personality traits (e.g., negative/positive urgency) and impulsive decision making styles observed in younger/middle age gamblers at baseline may negatively impact treatment response.

Besides, analysis in the total sample confirmed that higher discount rates predicted both the risk of relapses and dropout. In addition, the higher risk of relapses was also predicted by younger age and lower GD severity. Younger age has been described in previous studies as a sociodemographic risk factor for dropout in the case of GD due to, among other aspects, higher levels of impulsivity [81]. In addition, the association between a lower severity of GD and a higher risk of dropout has also been described by other authors, who suggest that it could be because these individuals may experience less interference from the disorder and therefore be less motivated to adhere to treatment, or that they may receive significant treatment benefits at the beginning of treatment, which could lead them to drop out of it [81].

Finally, the path analysis confirmed the direct link between DD and treatment outcome, considering both relapses and dropout. Furthermore, GD severity emerged as a crucial variable mediating the relationship between age, personality traits and DD and CBT outcome. Hence, younger patients, with higher values in delay discount rates, and specific personality traits (i.e., higher scores in novelty seeking, harm avoidance, and self-transcendence, and lower scores in reward dependence and cooperativeness) showed more severe GD symptoms, which in turn increase the likelihood of poor treatment outcome. These results contribute to the identification of the clinical profile of a gambler who is at risk of dropout and relapses, who is younger, with impulsive traits and poor decision making, and therefore presents more severe symptoms. Identifying specific factors underlying GD severity and poor response to treatment in younger patients is crucial to adapt personalized and more effective interventions. In this vein, tools that complement CBT, including new technologies [82], might help address certain underlying factors which are usually difficult to change, such as impulsivity or anger expression.

This is the first study that defines DD as an essential factor in the treatment response of individuals with GD that may interfere with it to a significant extent. However, the current findings should be interpreted considering some limitations. First, the majority of the patients who sought treatment for GD were male, which is consistent with previous reports [57,83,84]. To avoid gender as a source of bias leading to incorrect conclusions, and given the gender differences in GD profiles and course [85,86], only males were included in this study. Therefore, the present results could not be extended to female GD patients. Another limitation is that impulsive choices were assessed only at baseline, which did not allow for the evaluation of possible changes in DD rates after treatment and their impact on it. Considering how aspects of impulsivity might change during treatment and post treatment requires additional investigation.

## 5. Conclusions

In conclusion, baseline discount rates were associated with GD severity and with emotional impulsive traits, specifically in GD patients of young/middle age and with preferences for strategic gambling. More impulsive choices at baseline predicted poor treatment outcomes, which are also influenced by age, personality traits and GD severity. A steeper discount rate directly predicted a higher risk of dropout or relapses, and indirectly affected treatment outcome by the mediation of GD severity. Consequently, it is essential to take into account subjects’ levels of choice impulsivity before treatment initiation in order to control a negative impact on treatment adherence.

## Figures and Tables

**Table 1:** Correlations between delay discount rate with the clinical profile at baseline.

		^1^ Group of Age			^2^ Gambling Preference		
		Younger	Middle	Older	Non-Strat	Strategic	Mixed
	〈	N = 31	N = 61	N = 41	N = 96	N = 18	N = 19
Age (years-old)		**0.391** ^**†**^	0.046	0.172	0.028	**0.357** ^**†**^	**0.297** ^**†**^
Onset of GD (years-old)		−**0.258** ^**†**^	−0.040	0.062	−0.032	0.193	0.177
Duration of GD (years)		**0.527** ^**†**^	0.039	−0.078	0.051	0.056	0.152
Debts due to gambling		0.113	−0.009	0.066	0.038	−0.007	−0.213
DSM-5 criteria for GD	0.753	−0.095	**0.254** ^**†**^	0.002	0.021	**0.408** ^**†**^	**0.279** ^**†**^
SOGS-total	0.705	**0.245** ^**†**^	**0.317** ^**†**^	0.131	0.169	**0.444** ^**†**^	**0.595** ^**†**^
SCL-90R GSI	0.978	−0.231	−0.044	−0.121	−0.227	**0.522** ^**†**^	−0.120
SCL-90R PST	0.978	−0.151	0.039	−0.057	−0.138	**0.533** ^**†**^	0.060
SCL-90R PSDI	0.978	−0.134	−0.031	−0.208	−0.214	**0.504** ^**†**^	−**0.336** ^**†**^
UPPS-P Lack premeditation	0.802	−0.132	0.112	0.133	0.032	**0.251** ^**†**^	**0.459** ^**†**^
UPPS-P Lack perseverance	0.794	−0.013	0.062	0.134	0.025	**0.450** ^**†**^	0.187
UPPS-P Sensation seeking	0.866	0.091	−0.156	−0.119	−0.151	−0.126	0.153
UPPS-P Positive urgency	0.918	**0.330** ^**†**^	**0.244** ^**†**^	−0.007	0.056	**0.345** ^**†**^	**0.350** ^**†**^
UPPS-P Negative urgency	0.837	**0.273** ^**†**^	0.197	−0.002	0.025	**0.387** ^**†**^	**0.381** ^**†**^
UPPS-P Impulsivity total	0.918	0.224	0.141	0.013	−0.009	**0.344** ^**†**^	**0.526** ^**†**^
TCI-R Novelty seeking	0.707	0.048	−0.068	0.092	0.039	0.105	−0.092
TCI-R Harm avoidance	0.708	−0.173	0.240 ^**†**^	0.017	0.003	**0.606** ^**†**^	0.068
TCI-R Reward dependence	0.704	0.148	−0.122	−0.040	0.001	−0.223	−0.078
TCI-R Persistence	0.877	−0.087	−0.221	−0.187	−0.161	−**0.431** ^**†**^	−0.194
TCI-R Self-directedness	0.819	−0.176	−0.133	−0.023	0.009	−**0.557** ^**†**^	−**0.280** ^**†**^
TCI-R Cooperativeness	0.761	0.019	−**0.278** ^**†**^	−0.232	−0.181	−**0.450** ^**†**^	−0.193
TCI-R Self-transcendence	0.829	0.041	−0.142	−0.175	−0.181	−**0.253** ^**†**^	0.056

Note. ^1^ Age groups: younger (18–34 years old), middle (35–50 years old) and older (51–80 years old). ^2^ Partial correlations adjusted by age. 〈: Cronbach’s-alpha in the study. ^†^ Bold: effect size within the ranges mild-moderate to high-large.

**Table 2:** Association of delay discounting with the CBT outcomes.

		Odds Ratio (OR)		Correlation (R)		
	Sample Size	Risk Dropout	Risk Relapse	Number Sessions	Number Relapses	Euros Relapses
^1^ Groups of age						
Younger	31	**1.89** *	0.86	−**0.241** ^**†**^	−0.058	0.040
Middle	61	1.37	**1.59** *	−0.160	0.133	0.101
Older	41	1.41	1.26	−0.153	0.074	−0.010
^2^ Gambling preference						
Non strategic	96	**1.70** *	1.22	−0.206	0.035	−0.053
Strategic	18	0.62	**3.01** *	0.207	**0.425** ^**†**^	**0.255** ^**†**^
Mixed	19	**4.74** *	1.20	−**0.272** ^**†**^	−0.082	0.220

Note. ^1^ Age groups: younger (18–34 years old), middle (35–50 years old) and older (51–80 years old). ^2^ Results adjusted by age. * Bold: significant OR. ^†^ Effect size within the ranges mild-moderate to high-large.

**Table 3:** Predictive model for the CBT outcomes: logistic regression.

	Dropout						Relapses					
	B	SE	p	OR	95%CI OR		B	SE	p	OR	95%CI OR	
Age (years-old)	−0.043	0.022	**0.046** *	0.958	0.918	0.999	−0.024	0.017	0.170	0.976	0.944	1.010
Duration of GD (years)	0.007	0.040	0.852	1.008	0.931	1.090	−0.024	0.035	0.496	0.976	0.911	1.046
DSM-5 criteria baseline	−0.331	0.169	**0.049** *	0.718	0.516	0.999	−0.024	0.142	0.867	0.976	0.739	1.289
SCL-90R GSI baseline	0.166	0.498	0.739	1.180	0.445	3.131	0.435	0.397	0.273	1.546	0.709	3.368
UPPS-P total baseline	0.007	0.013	0.621	1.007	0.980	1.034	−0.014	0.011	0.214	0.986	0.965	1.008
Gambling preference	−0.219	0.551	0.691	0.803	0.273	2.368	−0.081	0.445	0.855	0.922	0.386	2.205
Delay discounting	0.441	0.186	**0.018** *	1.554	1.080	2.235	0.302	0.144	**0.036** *	1.352	1.020	1.792

Note. B: unstandardized coefficient. SE: standard error. OR: odds ratio. 95%CI: 95% confidence interval. * Bold: significant parameter. Gambling preference: 0 = non-strategic gambling and 1 = strategic or mixed. Sample size: n = 133.

## Supplementary Materials

The following supporting information can be downloaded at: https://www.mdpi.com/article/10.3390/jcm11061611/s1, Table S1: SEM: direct, indirect and total effect tests.
