# Supplementary material for: Delay Discounting in Gambling Disorder: Implications in Treatment Outcome
Source: J Clin Med. 2022 Mar 14;11(6):1611. doi: 10.3390/jcm11061611 (PMC8955705; doi:10.3390/jcm11061611)
Supplement: Supplementary file 1 [file jcm-11-01611-s001.zip › jcm-1561144-supplementary.pdf]

Table S1. (Supplementary) SEM: direct, indirect and total effect tests.

Direct Effects		Coeff	SE	z-stat	p	Std. Coeff
Structural						
TCI_Novelty seeking	Age	-0.158	0.084	-1.87	.049	-0.156
	Personality	1.000	(constrained)			0.154
TCI_Cooperativeness	Delay_discounting	-1.496	0.596	-2.51	.012	-0.160
	Personality	-4.014	1.037	-3.87	<.001	-0.540
TCI_Self-transcendence	Age	0.387	0.080	4.84	<.001	0.349
	Personality	1.671	0.645	2.59	.010	0.234
SOGS-total	Delay_discounting	0.409	0.150	2.73	.006	0.216
	Age	-0.050	0.018	-2.8	.005	-0.212
	Personality	0.453	0.152	2.98	.003	0.301
SCL-90R GSI	Delay_discounting	-0.054	0.025	-2.19	.028	-0.137
	Personality	0.185	0.045	4.08	<.001	0.594
CBT Bad outcome	SOGS-total	0.037	0.014	2.67	.008	0.222
	Delay_discounting	0.070	0.027	2.64	.008	0.221
	Age	0.000	(no path)			0.000
	Personality	0.000	(no path)			0.000
Measurement						
TCI_Harm avoidance	Personality	2.309	0.987	2.34	.019	0.286
TCI_Reward dependence	Personality	-1.929	0.659	-2.93	.003	-0.300
TCI_Persistence	Personality	-0.929	0.897	-1.04	.300	-0.091
TCI_Self-directedness	Personality	-9.698	2.015	-4.81	<.001	-1.000
Impulsivity (UPPS-total)	Personality	6.391	1.549	4.13	<.001	0.622
Gambling preference	Personality	0.047	0.020	2.3	.022	0.206
**Indirect effects**		**Coeff**	**SE**	**z-stat**	p	Std.Coeff
CBT Bad outcome	SOGS-total	0.000	(no path)			0.000
	Delay_discounting	0.015	0.008	1.91	.056	0.048
	Age	-0.002	0.001	-1.93	.053	-0.047
	Personality	0.017	0.008	1.99	.047	0.067
**Total effects**		**Coeff**	**SE**	**z-stat**	p	Std.Coeff
Structural						
TCI_Novelty seeking	Age	-0.158	0.084	-1.87	.049	-0.156
	Personality	1.000	(constrained)			0.154
TCI_Cooperativeness	Delay_discounting	-1.496	0.596	-2.51	.012	-0.160
	Personality	-4.014	1.037	-3.87	<.001	-0.540
TCI_Self-transcendence	Age	0.387	0.080	4.84	<.001	0.349
	Personality	1.671	0.645	2.59	.010	0.234
SOGS-total	Delay_discounting	0.409	0.150	2.73	.006	0.216
	Age	-0.050	0.018	-2.8	.005	-0.212
	Personality	0.453	0.152	2.98	.003	0.301
SCL-90R GSI	Delay_discounting	-0.054	0.025	-2.19	.028	-0.137
	Personality	0.185	0.045	4.08	<.001	0.594

CBT Bad outcome	SOGS-total	0.037	0.014	2.67	.008	0.222
	Delay_discounting	0.086	0.027	3.23	.001	0.269
	Age	-0.002	0.001	-1.93	.053	-0.047
	Personality	0.017	0.008	1.99	.047	0.067
Measurement						
TCI_Harm avoidance	Personality	2.309	0.987	2.34	.019	0.286
TCI_Reward dependence	Personality	-1.929	0.659	-2.93	.003	-0.300
TCI_Persistence	Personality	-0.929	0.897	-1.04	.300	-0.091
TCI_Self-directedness	Personality	-9.698	2.015	-4.81	<.001	-1.000
Impulsivity (UPPS-total)	Personality	6.391	1.549	4.13	<.001	0.622
Gambling preference	Personality	0.047	0.020	2.3	.022	0.206

Note. Coeff: un-standardized coefficient. SE: standard error. Std.Coeff: standardized coefficient. Sample size: n=133.
